# Expression of Glycogen Phosphorylase Isoforms in Cultured Muscle from Patients with McArdle's Disease Carrying the *p.R771PfsX33 PYGM* Mutation

**DOI:** 10.1371/journal.pone.0013164

**Published:** 2010-10-05

**Authors:** Gisela Nogales-Gadea, Emma Mormeneo, Inés García-Consuegra, Juan C. Rubio, Anna Orozco, Joaquin Arenas, Miguel A. Martín, Alejandro Lucia, Anna M. Gómez-Foix, Ramon Martí, Antoni L. Andreu

**Affiliations:** 1 Departament de Patologia Mitocondrial i Neuromuscular, Institut de Recerca, Hospital Universitari Vall d'Hebron, Barcelona, Spain; 2 Departament de Bioquímica i Biología Molecular, Facultat de Biologia, Universitat de Barcelona, Barcelona, Spain; 3 Centro de Investigación, Hospital Universitario 12 de Octubre, Madrid, Spain; 4 Universidad Europea de Madrid, Madrid, Spain; 5 Spanish Network for Research in Rare Diseases (CIBERER), Instituto de Salud Carlos III, Madrid, Spain; 6 Diabetes and Associated Metabolic Disorders (CIBERDEM), Instituto de Salud Carlos III, Madrid, Spain; Instituto de Biomedicina de Valencia, CSIC, Spain

## Abstract

**Background:**

Mutations in the *PYGM* gene encoding skeletal muscle glycogen phosphorylase (GP) cause a metabolic disorder known as McArdle's disease. Previous studies in muscle biopsies and cultured muscle cells from McArdle patients have shown that *PYGM* mutations abolish GP activity in skeletal muscle, but that the enzyme activity reappears when muscle cells are in culture. The identification of the GP isoenzyme that accounts for this activity remains controversial.

**Methodology/Principal Findings:**

In this study we present two related patients harbouring a novel *PYGM* mutation, *p.R771PfsX33*. In the patients' skeletal muscle biopsies, *PYGM* mRNA levels were ∼60% lower than those observed in two matched healthy controls; biochemical analysis of a patient muscle biopsy resulted in undetectable GP protein and GP activity. A strong reduction of the *PYGM* mRNA was observed in cultured muscle cells from patients and controls, as compared to the levels observed in muscle tissue. In cultured cells, *PYGM* mRNA levels were negligible regardless of the differentiation stage. After a 12 day period of differentiation similar expression of the brain and liver isoforms were observed at the mRNA level in cells from patients and controls. Total GP activity (measured with AMP) was not different either; however, the active GP activity and immunoreactive GP protein levels were lower in patients' cell cultures. GP immunoreactivity was mainly due to brain and liver GP but muscle GP seemed to be responsible for the differences.

**Conclusions/Significance:**

These results indicate that in both patients' and controls' cell cultures, unlike in skeletal muscle tissue, most of the protein and GP activities result from the expression of brain GP and liver GP genes, although there is still some activity resulting from the expression of the muscle GP gene. More research is necessary to clarify the differential mechanisms of metabolic adaptations that McArdle cultures undergo *in vitro*.

## Introduction

Glycogen phosphorylase (GP) is a finely regulated key enzyme in the metabolism of glycogen. Its 1,4-α-glucan-phosphate-glucosyltransferase activity catalyzes a limiting step in glycogenolysis, i.e. glycogen phosphorolysis to form glucose-1-P [1]. There are three different GP isoenzymes encoded by three different genes, apparently evolved from a common ancestral gene, i.e. *PYGB* (brain) *PYGL* (liver), and *PYGM* (skeletal muscle) [2].

In 1959, lack of muscle GP was identified as the cause of a glycogenolytic defect confined to the skeletal muscles [3], [4]. The clinical features of this disorder, known as McArdle's disease or glycogenolysis type V had first been described a few years earlier by Brian McArdle [5], and encompass exercise intolerance with reversible acute crises of premature fatigue, myalgia and contractures, sometimes accompanied by severe rhabdomyolysis and myoglobinuria; these episodes are triggered by static or isometric muscle contractions as well as by dynamic, strenuous exercises such as running [6].

Since the publication of the first pathogenic *PYGM* mutations in 1993 [7], [8], a growing allelic heterogeneity of the *PYGM* gene has been reported, with more than 100 mutations known to cause McArdle's disease [9]. A stop-codon mutation, *p.R50X*, is commonly encountered in European and American patients with an allelic frequency ranging from 81% to 43% in different populations. However, most mutations are private, i.e. reported in a single patient or in the members of the same family [9]. Moreover, studies on skeletal muscle cDNA resulted in: (i) the identification of mutations that genomic direct analysis failed to detect, and (ii) understanding the effect of *PYGM* mutations in gene expression [10]. An RNA surveillance mechanism known as ‘nonsense mediated mRNA decay’ (NMD), reduces the mRNA levels of those transcripts that contain nonsense and frameshift mutations [11]. Our previous results support the notion that NMD is a common acting mechanism among McArdle patients, with 92% of them showing a reduced amount of *PYGM* mRNA levels [12].

GP activity in muscle biopsies and cultured muscle cells from McArdle patients has previously been studied. No detectable GP activity is observed in muscle biopsies from patients; however, cultured muscle cells derived from the same biopsies did present GP activity [13], [14], [15], [16]. It has also been described in regenerative fibers from McArdle patients [17]. This phenomenon was described as “the mystery of the reappearing enzyme” [17], [18], although it is not clear which specific GP isoform accounts for this activity, i.e. brain isoform [15], brain and liver isoform [16] vs. skeletal muscle isoform [13], [14].

In this study we have characterized the molecular alterations produced by a novel frameshift *PYGM* mutation (*p.R771PfsX33*), at the mRNA and protein levels, in the skeletal muscle biopsy of two related McArdle patients. In addition, we also cultured skeletal muscle cells, determined the expression of the different GP isoforms, and characterized the glycogen metabolism by determining GP activity, glycogen synthase (GS) activity and glycogen content.

## Methods

### Ethics Statement

Written informed consent was obtained from all individuals. The study was approved by the institutional review board of the University Hospital *12 de Octubre* and was carried out in accordance with the Declaration of Helsinki for Human Research.

### Subjects

We report two Caucasian brothers (index case P1, and P2), aged 43 and 51 years, from a small village in southern Spain, with family history of consanguinity but not of neuromuscular diseases. They both presented the four cardinal features of the disease [6]: (i) exercise intolerance since childhood; (ii) high serum levels of creatine kinase (CK) activity, even in basal conditions (672 U·l^−1^ and 344 U·l^−1^ at the moment of study, after 2 resting days, normal <170 U·l^−1^); (iii) previous episodes of hyper-CK-emia (∼7,000 and 10,000 U·l^−1^) plus myoglobinuria after intense exercise, indicating marked rhabdomyolysis; and (iv) the ‘second wind’ phenomenon, depicted by a sudden, marked improvement tolerance to aerobic dynamic exercise (notably, brisk walking) after ∼8–10 minutes of exercise or after a short period of rest [19]. Their peak oxygen uptake (VO_2peak_) measured during incremental cycle-ergometer testing was very low (12.5 and 13.0 ml O_2_/kg/min), barely above the limits for independent living, which reflects a markedly decreased muscle oxidative capacity, another common feature of the disease [20]. Two sex- and age-matched healthy Spanish volunteers recruited for the study (C1, C2) served as controls (Table 1). Their VO_2peak_ was 36 and 38 ml O_2_/kg/min.

**Table 1:** Subjects' information.

Subject	Age*	Sex	*PYGM* genotype	*PYGM* mRNA (%)
P1	44	Male	*p.R771PfsX33/p.R771PfsX33*	40
P2	55	Male	*p.R771PfsX33/p.R771PfsX33*	32
C1	43	Male	wild type/wild type	94
*C2*	46	Male	wild type/wild type	107

*Age at the time the skeletal muscle biopsy was collected. GenBank reference sequence was NP_005600.1. Expression values were calculated considering 100% the mean results obtained in the controls (C1, C2).

### Biochemical and genetic analyses

McArdle's disease was suspected on clinical bases in the index case (P1). A muscle biopsy was obtained to assess glycogen phosphorylase activity, which was undetectable. Thus, the diagnosis was confirmed genetically by *PYGM* sequencing in P1 and his brother (P2), who was also clinically affected (see above). *PYGM* gene was sequenced as follows: DNA was isolated from whole blood using a standard phenol-chloroform method (Nucleon BACC-2, GE healthcare Europe GMBH, Chalfont St. Giles, UK). We amplified the coding sequence of the entire *PYGM* gene by polymerase chain reaction (PCR) in 14 fragments, using the primers described by Kubisch et al [21]. For PCR analysis and sequencing, we followed the steps described elsewhere [22]. In order to perform a more exhaustive screening, we performed *PYGM* amplification of cDNA samples (see below), with a different set of primers [12].

### Muscle samples

We obtained a *biceps* biopsy from P1 for diagnostic purposes (GP activity assessment) and immunoblotting. Once McArdle's disease was confirmed, a new *biceps* biopsy was obtained from P1, as well as from P2, C1 and C2, for research purposes. Each biopsy specimen was divided in two fragments, for myoblast isolation and for RNA extraction.

### Muscle cell culture, immunohistochemistry and electron microscopy

Myoblast cell populations were isolated from muscle biopsies using the explant culture technique [23]. Myoblasts were grown in DMEM/M-199 medium, 3∶1, with 10% fetal bovine serum (FBS), 10 µg/ml insulin, 2 mM glutamine, 25 ng/ml fibroblast growth factor and 10 ng/ml epidermal growth factor. Confluent cells were induced to differentiate for 7 or 12 days by removing fibroblast and epidermal growth factors. Twenty-four hours before the metabolic experiments, cells were depleted of insulin and FBS. For immunohistochemistry analyses, cells grown on coverslips were fixed in 4% paraformaldehyde in PBS, then washed with PBS followed by 3 sequential treatments with PBS containing: (1) 20 mM glycine, (2) 0.1% Triton X-100 and (3) 1% BSA. Next, coverslips were incubated with a rabbit anti-desmin antibody (AB907 Millipore, Billerica, MA, USA). The primary antibody was detected with an Alexa Fluor-488 goat anti-rabbit antibody (Molecular Probes, Eugene, OR, USA). Both primary and secondary antibodies were diluted in blocking solution. Nuclei were stained with Hoescht (1 µg/ml) after secondary antibody incubation. After staining, samples were mounted and analyzed with a Leica TCS SP2 confocal microscope. For electron microscopy, cells were fixed in 2.5% glutaraldehyde in 0.1 M phosphate buffer (PB) (pH 7.4), then cells were scraped in PB and post-fixed with 1% osmium tetroxide in PB containing 0.8% potassium ferricyanide at 4°C. Then cells were dehydrated in acetone, infiltrated in Epon resin for 2 days, embedded in the same resin and polymerised at 60°C during 48 h. Ultrathin sections were obtained using a Leica Ultracut UCT ultramicrotome and mounted on Formvar-coated cooper grids. They were stained with 2% uranyl acetate in water and lead citrate. Then sections were observed in a JEM-1010 electron microscope (Jeol, Tokyo, Japan). The diameter size of glycogen granules was measured with AnalySIS (Soft Imaging System, Olympus, Hamburg, Germany). The amount and diameter of glycogen particles were measured in images of myotubes obtained at x 30,000 magnification. Five cells from C1 and P1 and three different cell areas per cell were examined.

### cDNA synthesis

Total RNA was extracted from the biopsies and cultured cells using Trizol® reagent (Invitrogen, Carlsbad, USA). To eliminate any traces of DNA, RNA was treated with the DNAse I, amplification grade (Invitrogen, Carlsbad, USA). The RNA concentration and quality was measured with NanoChips, using the Boanalyzer 2100 system and the 2100 Expert Software version B.02.02 (Agilent, Santa Clara, USA). cDNA was synthesized from muscle RNA using the high-capacity cDNA archive kit (Applied Biosystems, Foster City, USA), which uses random primers.

### Real Time PCR analysis

GP mRNA levels were quantified by real-time PCR, using TaqMan fluorogenic probes in a 7,500 Real Time PCR System (Applied biosystems, Foster City, USA). We used a probe located in exons 6–7 for the detection of *PYGM* mRNA (Hs00194493_m1), a probe located in exons 5–6 for *PYGB* mRNA (Hs00267875_m1), and a probe located in exons 3–4 for *PYGL* mRNA (Hs00161132_m1). Results were normalized to cyclophilin A (*PPIA*) mRNA levels (probe Hs0099999904_m1). The real time PCR was performed as described elsewhere [12]. When comparing gene expression levels between C2 skeletal muscle biopsy specimen and its corresponding cell culture, we used the amount of total RNA per well to normalize the results. Similar efficiencies of amplification were observed for the *PGYM*, *PYGB*, *PYGL* and *PPIA* cDNAs.

### Enzyme activity and glycogen assays

GP activity was measured in the muscle biopsy of patient P1 following the standard procedure [24]. In order to measure GS and GP activities in the cultured cells, we used 100 µl of homogenization buffer (10 mM Tris-HCl pH 7.0, 150 mM KF, 15 mM EDTA, 600 mM sucrose, 15 mM 2-mercaptoethanol, 17 µg/l leupeptin, 1 mM benzamidine and 1 mM phenylmethylsulfonyl fluoride) to scrape frozen plates containing the cell monolayers prior to sonication. The homogenates were used to determine enzyme activities. GP activity was determined by the incorporation of [U-^14^C] glucose 1-phosphate into glycogen in the absence (activated GP) or presence (total GP) of the allosteric activator AMP (1 mM) [25]. GS activity was measured in the absence (activated GS) or presence (total GS) of 10 mM glucose 6-P as described [26]. Aliquots of the homogenates were used to measure protein concentration. Glycogen was extracted from cell monolayers as previously described [27] and released glucose quantified with the Glucose (HK) assay kit (Sigma Aldrich, Saint Louis, USA).

### Immunoblotting analysis

Skeletal muscle biopsies were homogenated in 50 mM Tris-HCl pH 7.5, 150 mM NaCl and 1% SDS. Cell extracts prepared as described above were mixed 1∶1 with a homogenization buffer consisting of 50 mM Tris-HCl pH 7.5, 150 mM NaCl, 1 mM EDTA, 1 mM phenylmethylsulfonyl fluoride, 1 mM NaF, 1 mM Na3VO4, 2 µg/µl benzamidine, 2 µg/µl leupeptin, 1% (v/v) Nonidet P40, and 1 mM dithiothreitol. Lysates were then gently rocked for 60 min at 4°C, and stored at −80°C until analysis. Protein was resolved in 10% SDS-PAGE. Antibodies against muscle GS (3893, Cell Signaling, Danvers, USA), brain/muscle GP (4GP31/17B6, HyTest, Turku, Finland), muscle GP in biopsy (AB63158, Abcam, Cambridge, UK) and for muscle GP in cell cultures (LS-A2284 MBL International Corporation, Woburn, USA), brain GP (BB-1F9:SC-81751, Santa Cruz Biotechnology, CA, USA) and liver GP (HPA000962, Sigma, St. Louis, USA) were utilized. Horseradish peroxidase-conjugated secondary antibodies were used and membranes were developed with ECL-Plus (GE Healthcare, Buckinghamshire, England). Bands were obtained with a LAS-3000 (FujiFilm, Tokyo, Japan) and quantified with NIH image J, version 1.37 analysis software (Scion image, NIH). α-Actin (C-terminal anti-actin antibody, Sigma, Saint Louis, USA) and α-tubulin (Sigma, Saint Louis, USA) were used as housekeeping proteins.

### Statistical analysis

We performed all statistical analysis with the SPSS package (SPSS 12.0, Chicago, IL, USA). The Mann Whitney's *U* test was used to analyze differences between the two groups (patients and controls).

## Results

### Genetic analysis

**Figure 1:**
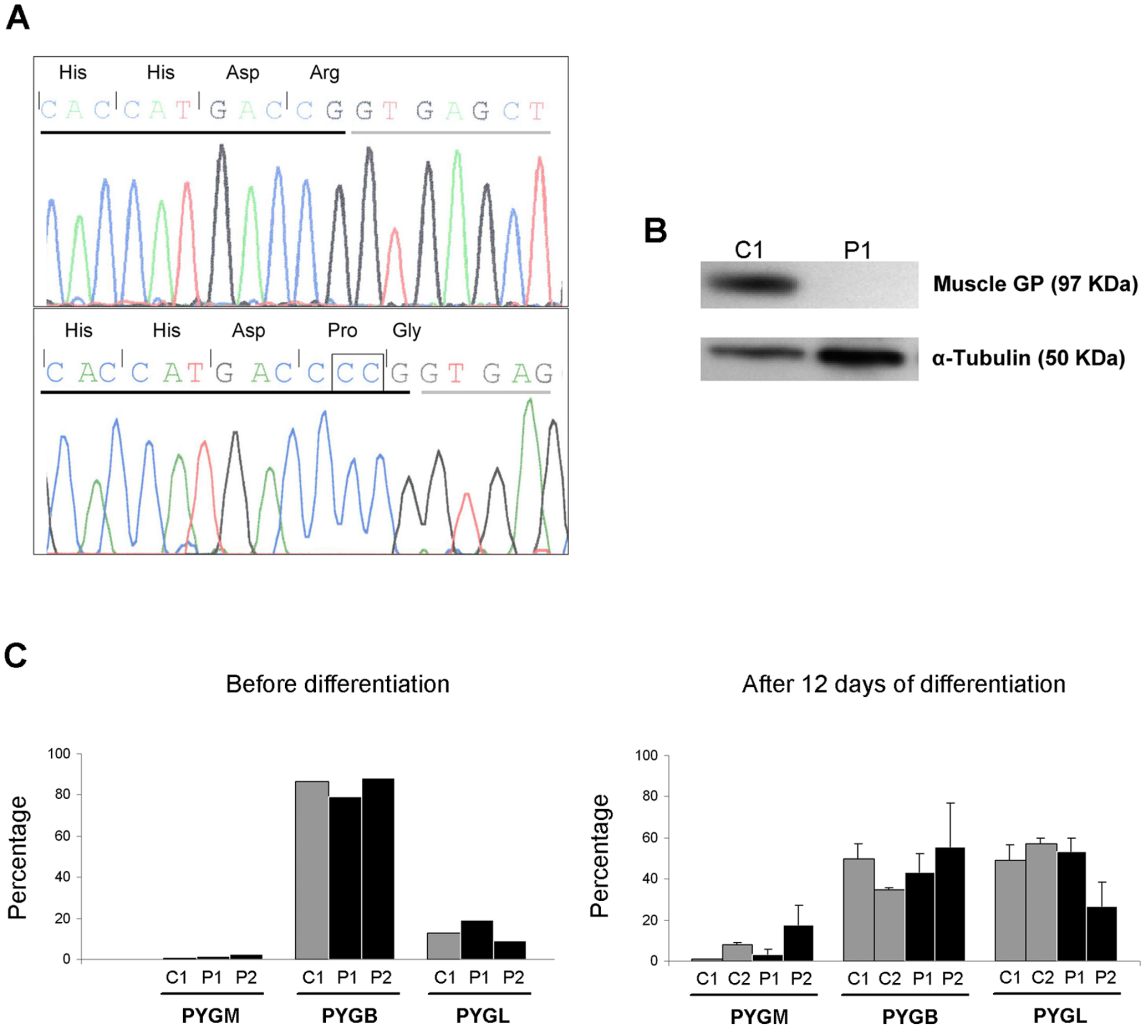
Molecular effects of *c.2310_2311dupCC* mutation and GP isoforms expression in muscle cells. (A) Electropherogram of *PYGM* exon 18 in control C1 (top) and patient P1 (bottom). The square shows the *c.2310_2311dupCC* mutation. Exonic nucleotides are underlined in black; intronic nucleotides are underlined in grey. Encoded amino acids are indicated (including wild type Arg, and mutant Gly, encoded by codons formed in an exon-exon junction). (B) Immunoblotting showing muscle GP and α-tubulin bands in muscle biopsy homogenates from control C1 and patient P1. (C) Relative contribution of each gene (*PYGM*, *PYGB* and *PYGL*), to the total amount of GP mRNA in undifferentiated and 12 day differentiated cultured muscle cells. Bars represent the result of a single experiment for undifferentiated cells, or mean ± SD of two independent experiments for 12 days differentiated cells. Percentages were calculated as [*PYG(x)* mRNA x 100/(*PYGB* mRNA + *PYGL* mRNA + *PYGM* mRNA)], using values normalized for the *PPIA* mRNA. In C1 and C2 skeletal muscle (not shown), *PYGB* mRNA and *PYGL* mRNA were negligible (<0.5%).

Sequencing of the PCR products spanning the entire *PYGM* gene coding region revealed the presence of a homozygous CC duplication in the exon 18 (*c.2310_2311dupCC*) in both patients (Figure 1A). This mutation changes arginine to proline in the position 771 of the protein and alters the open reading frame predicting a premature stop codon 33 amino acid residues downstream (*p.R771PfsX33*). No other mutations were found in the coding sequence or flanking intronic regions of the *PYGM* gene, nor in the cDNA. The nomenclature of the mutation is referred to the GenBank entries NM_005609.1 (cDNA) and NP_005600.1 (protein).

### Differential expression of GP genes in skeletal muscle and cultured cells

Total GP activity was measured in skeletal muscle biopsy from patient P1 and was undetectable. GP activity was not analyzed in P2 muscle because the diagnostic was confirmed in P2 by *PYGM* gene sequencing. Muscle *PYGM* mRNA levels of P1 and P2 were reduced to 40% and 32% respectively, as compared to the mean of C1 and C2 *PYGM* mRNA levels (Table 1). A specific antibody against muscle GP was used to detect this isoform in C1 and P1 muscle homogenates, resulting in the expected 97 kDa muscle GP band in C1, which was absent in P1 (Figure 1B).

**Figure 2:**
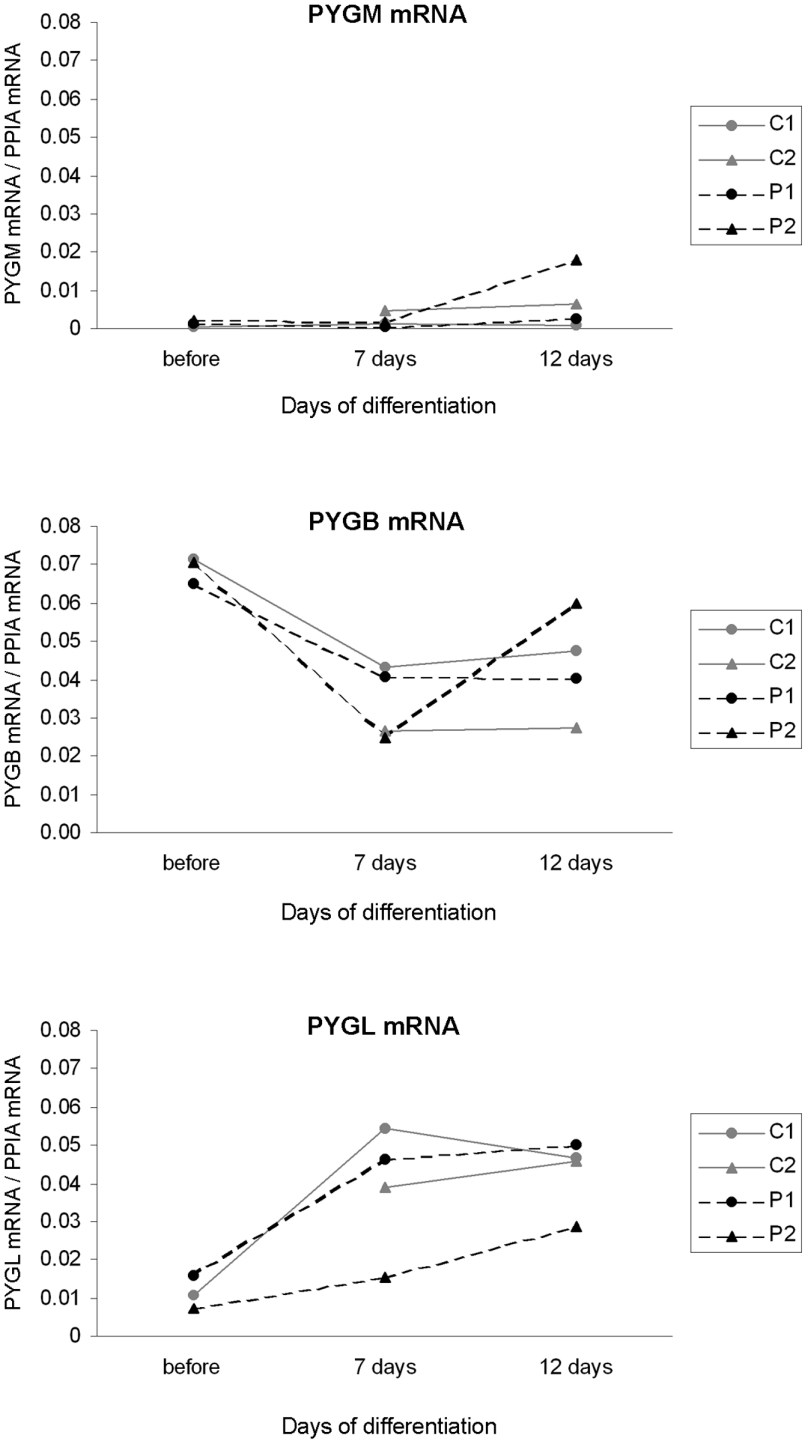
mRNA quantification of GP genes at 0, 7 and 12 days of differentiation. Results from a single experiment (before differentiation; C1, P1 and P2), or mean of two independent experiments (7-day and 12-day differentiation, C1, C2, P1 and P2) are depicted.

While virtually all the mRNA found in C1 and C2 skeletal muscle was the product of the expression of *PYGM* (>99%), this gene poorly contributed (between 0.6 – 2.5%) to the total GP mRNA in undifferentiated cultured cells, either from patients or controls, and only reached moderate contribution (17%) in 12 days differentiated P2 cells (Figure 1C). When referred to total RNA, *PYGM* mRNA levels were approximately 27-fold higher in C2 muscle than in cultured cells. By contrast, *PYGL* mRNA was 17-fold higher in cultured cells, while *PYGB* mRNA levels were similar in muscle tissue and cultured cells (data not shown). The relative contribution of each gene (*PYGB* and *PYGL*) to the total amount of GP mRNA in muscle, undifferentiated and differentiated cultured cells was markedly different (Figure 1C). Before differentiation, the major contributor in cell culture of C1, P1 and P2 was *PYGB* gene, while 12 days after differentiation both *PYGB* and *PYGL* genes contributed equally to the total GP mRNA pool in C1, C2 and P1, and moderately higher *PYGB* mRNA was found in P2. However, no significant differences between patients and controls were observed in the relative contributions of each gene in cultured cells. Figure 2 shows the changes in the mRNA levels, product from the three different genes, over a period of differentiation of 12 days. The inspection of the data did not show obvious differences in the distribution of the mRNA levels between patients and controls for any of the GP isoforms. Overall, *PYGB* mRNA had tendency to decrease with the time in culture, while *PYGL* mRNA seemed to increase. *PYGM* mRNA did not show a clear tendency, except for a moderate increase in 12 day differentiated P2 cells. In absolute amounts, this variation was low when compared to that observed for *PYGB* and *PYGL* mRNAs, due to the minute amounts of *PYGM* mRNA detected in cultured cells as compared to the other isoforms.

### Cell morphology

**Figure 3:**
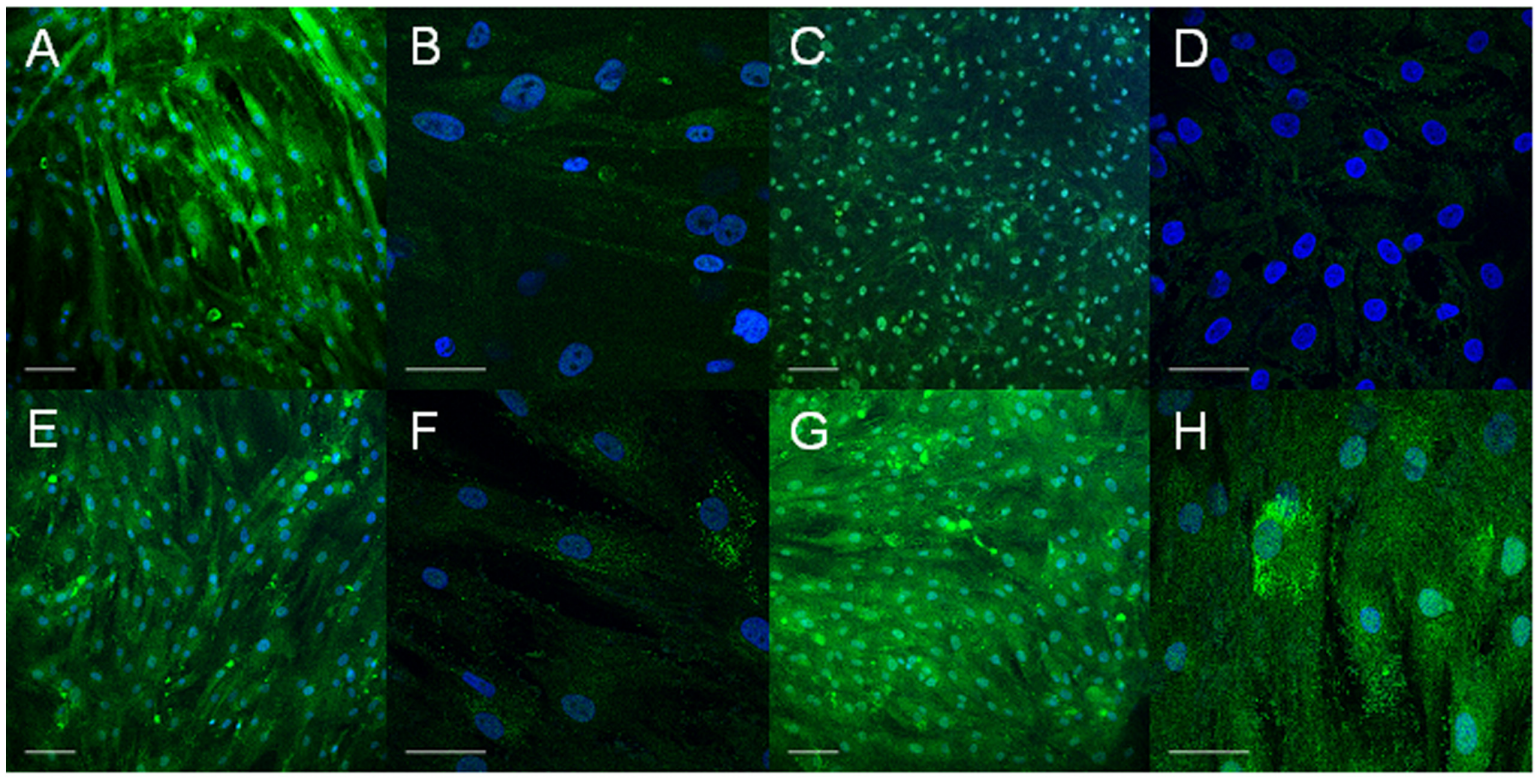
Immunostaining of myotube cultures with the muscle-specific marker desmin. Myotubes at 7 days post-differentiation were immunostained with desmin antibody and then cell nuclei were stained with Hoescht. Representative immunofluorescence micrographs of skeletal muscle cultures are shown: (A,B,E,F) C1, (C,D) P1 and (G,H) P2. In A, C, E and G white bars represent 100 µm. In B, D, F and H white bars represent 50 µm.

Muscle cells were prevalent in the control C1 and patients P1 and P2 cultures as assessed by immunostaining for desmin, a muscle-specific intermediate filament protein. As shown in Figure 3, at day 7 post-differentiation, most of the Hoescht-stained nuclei are observed in desmin-labeled cells. Noteworthy, in cultures from patient P1, unfused mononucleated myoblasts were abundant, whereas in control C1 and patient P2, myotubes prevailed. Cell morphology was similar at day 12 post-differentiation, although muscle cells appeared less delineated in the three cell cultures (data not shown).

### Enzyme activities and glycogen content in the cell cultures

**Figure 4:**
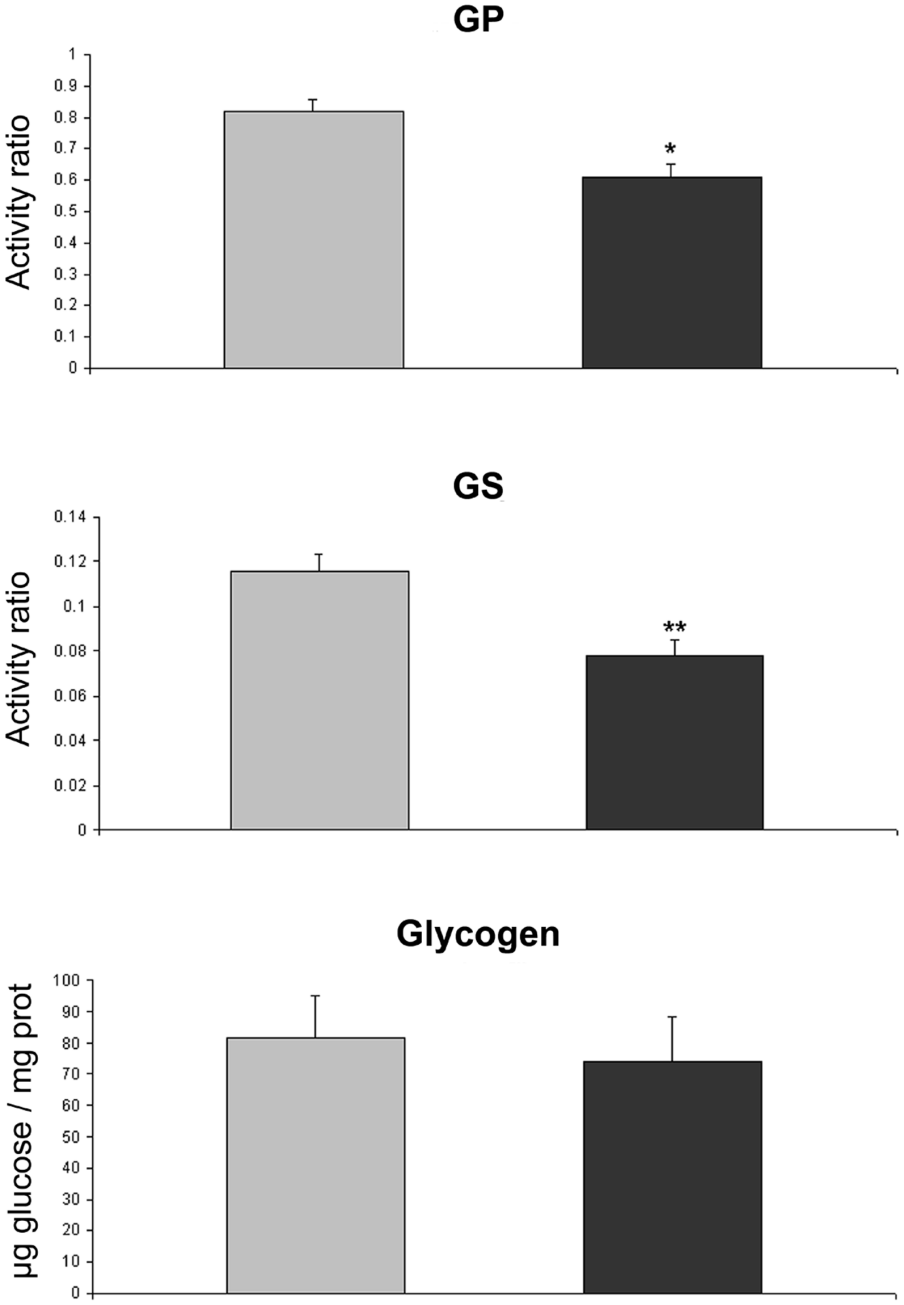
Glycogen phosphorylase activity ratio (active/total), glycogen synthase activity ratio (active/total) and glycogen content. The results represent values for controls (C1, C2) and patients (P1, P2), after 7 days of differentiation. Values are mean ± SEM. The significance of the difference versus controls is: *p<0.01 and **p<0.001. Grey bars represent controls and black bars patients.

**Figure 5:**
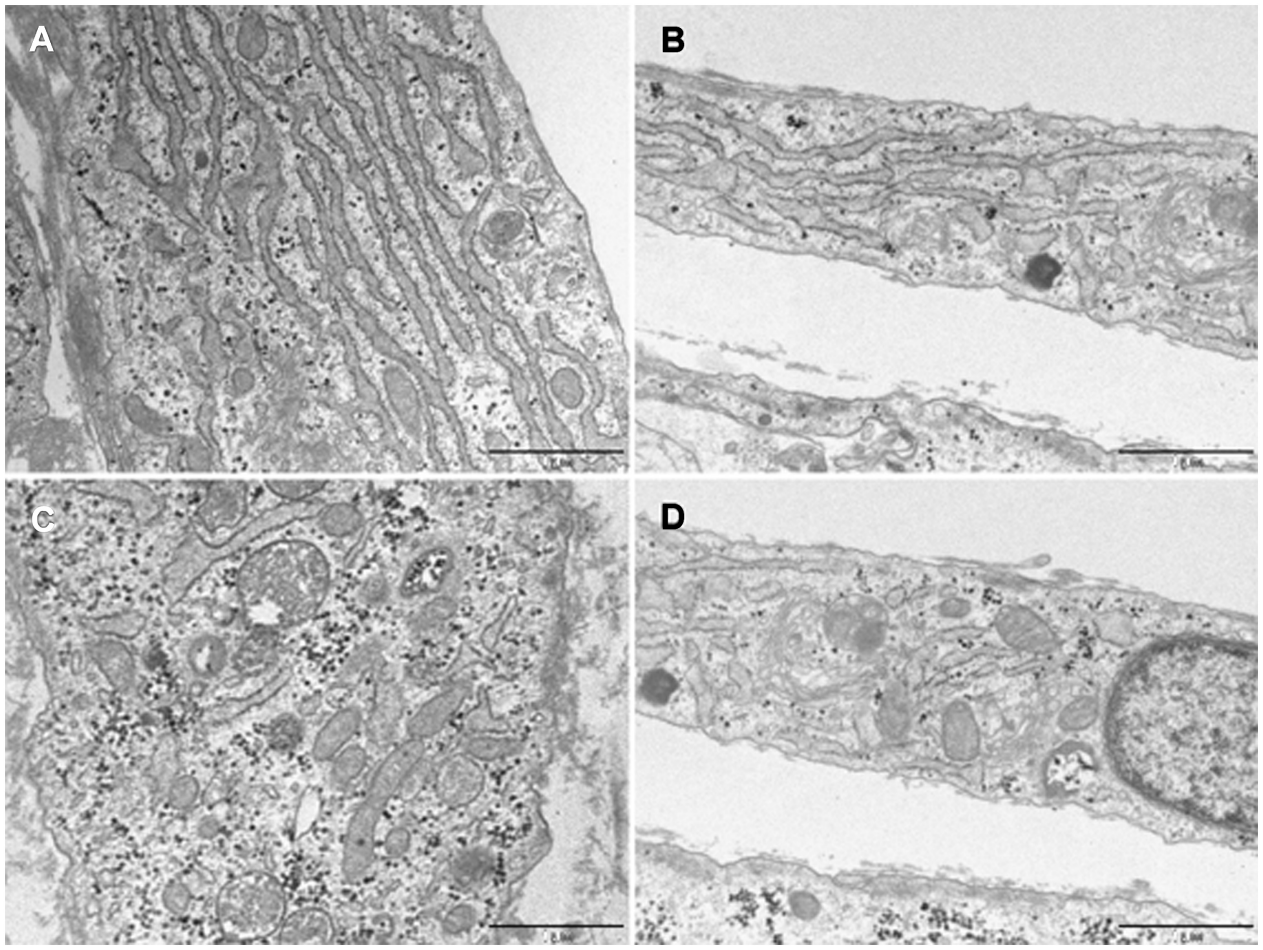
Electron microscopic analysis of glycogen stores. Electron micrograph of myotubes cultured from (A,C) control C1 and (B,D) patient P1, at 10 days post-differentiation. Glycogen granules and clusters appear as black dense particles in the cytoplasm. Bar represents 1 µm. Images were obtained at x 30,000.

We performed biochemical analyses of the cell cultures after 7 days of differentiation. No differences were found when comparing the total activity of GP (controls 136±8 munits/mg of protein and patients 121±12 munits/mg of protein) and GS (controls 22.0±2.5 munits/mg of protein and patients 20.4±1.6 munits/mg of protein) between patients and controls. However, the active forms of GP and GS activities were about 50% lower in the patients' cell cultures with statistical significance being reached (p*<*0.05). Consequently, the GP and GS activity ratios (active/total) (Figure 4) were also lower in the patients' cell cultures. The amount of glycogen in the patients' cell lines was not significantly different from that of control lines, although they had a tendency to a lower glycogen concentration (Figure 4). Electron microscopic evaluation of glycogen stores (Figure 5) showed that cells from patient P1 had a significantly (p = 2.86×10^−7^) lower number of glycogen granules per cell (45.91±11.35) than control C1 cells (153.0±52.42), but slightly larger granules of glycogen (27.19±8.98 nm) than control C1 cells (25.44±9.04 nm) (p = 0.001). In both cell types, glycogen granules and small glycogen clusters were scattered through the cytoplasm; additionally glycogen accumulation in large multigranular bodies was observed (not shown).

### Immunoblotting analysis in the cell cultures

**Figure 6:**
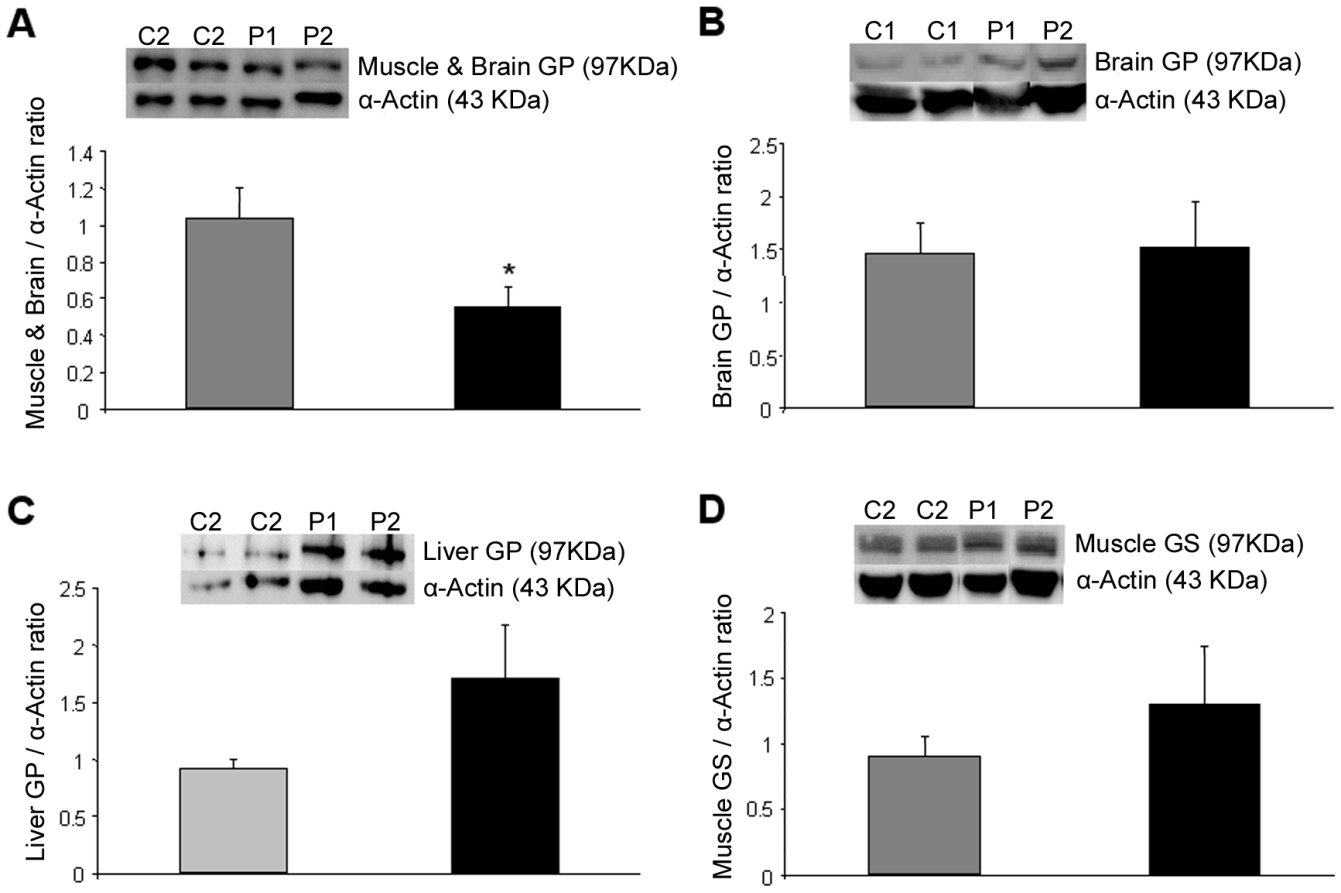
Immunoblotting analysis for brain/muscle GP, brain GP, liver GP and muscle GS proteins, of 7 days differentiated cell lines. Grey bars represent controls (C1, C2) and black bars patients (P1, P2). Anti-actin immunoblotting was performed as loading control. Representative images of brain/muscle GP (A), brain GP (B), liver GP (C) and muscle GS (D) are shown. Ratios of intensity of GP (A, B and C) and GS (D) bands compared to intensity of α-actin bands are shown as mean ± SEM. The significance of the difference versus controls is *p<0.05.

We determined the content of GP protein isoforms in the cell cultures after 7 days of differentiation (Figure 6). First, the brain/muscle GP protein level was assessed with an antibody that besides the brain GP protein recognizes the muscle GP protein when overexpressed in cultured myotubes [28]. We observed about 50% lower levels of brain/muscle GP isoforms relative to α-actin protein in the patients' lines. No signal was however detected when a muscle GP specific antibody was used (data not shown), likely due to the very low gene expression levels, as previously observed [28]. No statistically significant differences were detected when using brain GP or liver GP specific antibodies relative to α-actin protein, in either controls' or patients' cells, although a tendency to increase was observed in patient's cells. We found no significant differences in muscle GS protein content with a rising tendency in patient's cells also.

## Discussion

Allelic heterogeneity is a characteristic of *PYGM* genotypes in McArdle patients, as many mutations are only found in a single patient or in the members of the same family. Here, we have identified the mutation *p.R771PfsX33* in homozygosity in two brothers with McArdle's disease. This mutation alters the open reading frame and generates a premature stop codon, which predicts the loss of the final 72 amino acids of the C-terminal domain. These include codons 824 and 825, strongly conserved in 14 GP enzymes from different species [1]. Other mutations generating premature stop codons near codon 771 have been reported to cause McArdle's disease: *p.E779delE*, found in a Korean patient [29], and *p.C784X* [22] and *p.E797VfsX18* [30], both described in Spanish patients. The new mutation reported here should be added to the long list of pathogenic variations described in the *PYGM* gene.

We recently demonstrated absence of *PYGM* transcripts in the skeletal muscle of patients carrying nonsense and frameshift mutations, most likely due to mRNA degradation induced by NMD [12]. This is a critical process for normal cellular development; it protects the organism from deleterious dominant-negative or gain-of-function effects caused by truncated proteins translated from stable nonsense transcripts [31], [32]. Nagy and Maquat [33] originally predicted that any premature termination codon occurring 50 to 55 nucleotides downstream of the 3′ most exon-exon junction of a gene would bypass NMD and further studies showed that mutations located in different exonic regions of the same gene may exhibit different NMD responses [34], [35]. Since *p.R771PfsX33* fulfils the characteristics to escape NMD, we studied *PYGM* expression. The levels of *PYGM* transcripts in the skeletal muscle of our patients were around 30–40% of those of controls. This reduction is probably due to NMD, yet of a smaller magnitude than previously described for other frameshift and nonsense *PYGM* mutations, in which transcript levels as low as 1–5% were observed [12]. Interestingly, *PYGM* mRNA levels were similar in our two patients, in agreement with our previous findings showing more similar *PYGM* expression levels among related patients as compared to unrelated patients [12]. Thus, some unknown factors irrespective of the type of *PYGM* mutation could also affect the transcription of the gene or the stability of the transcript.

In contrast to the presence of substantial *PYGM* mRNA levels in patients' skeletal muscle, this GP isoform was undetectable by immunoblot in muscle biopsy from P1. This result is consistent with the absence of GP activity in P1 muscle, and might indicate an alteration in the translation of mutant mRNA or changes in the stability of the mutant protein. Translational control is mediated by regulatory proteins or noncoding RNAs that bind to the 3′- or 5′- UTR of target mRNAs [36]. The UTR structures can be missed through disease-causing mutations in coding regions [37], as previously observed [38]. Similarly, *p.R771PfsX33* could be changing the translation regulation of muscle GP in patients bearing this mutation.

Pioneer experimental studies have been performed over the last three decades to obtain and characterize McArdle patients' muscle and muscle cell cultures [13], [14], [15], [16], [17], [39]. These studies revealed that cultured muscle cells from McArdle patients have GP activity, even though they are derived from GP-negative muscle tissue. Interestingly, some regenerative fibres from patients also “recover” GP activity together with expression of fetal myosin [17]. Most of these studies were carried out before the *PYGM* gene was discovered and when the number of GP isoenzymes was unknown. This explains, at least partially, the diversity of findings on GP expression in McArdle muscle cell cultures and the lack of reports on possible expression of other isoforms. The development of tools that allow us to distinguish GP mRNA isoforms prompted us to perform *in vitro* studies to characterize the GP gene expression pattern in samples with the *p.R771PfsX33* mutation. We only studied two patients harbouring the same mutation, but we believe that our results are of interest when considering the scarcity of available *in vitro* studies on McArdle's disease.

*PYGM* mRNA levels dropped drastically in muscle cell culture in all differentiation stages, as compared to those observed in control muscle *in vivo*. In fact, a transcriptome microarray analysis has shown that *PYGM* is among the top downregulated genes in human cultured myotubes compared to skeletal muscle biopsies [23]. In contrast, the *PYGL* messenger was increased in cell culture, while *PYGB* mRNA levels were similar to those observed in *in vivo* muscle. Therefore, the muscular isoenzyme is the only form that becomes strongly reduced in cell culture, and this accounts for the strong reduction of GP activity in muscle cultured cells as compared to muscle tissue previously reported [14], [16]. Additionally, the pattern of GP isoforms gene expression changes in muscle cell cultures along differentiation. This should be taken into account when cellular models are used for molecular and biochemical studies in McArdle's disease. In this regard, using microarray transcriptional analysis we have previously characterized time-dependent changes in the gene expression profile of cultured human myotubes mostly affecting contractile and apoptosis-related genes [40].

Immunoblotting experiments did reflect: (i) lower content of the brain/muscle GP protein in patients' than controls' muscle cultures, (ii) no significant differences but a tendency to increase when using the specific brain or liver GP antibodies, and (iii) no signal with the muscle GP-specific antibody in both cultured cell types, probably because the GP muscle isoform was below detection level. These data suggest that most of the protein we detected with the brain/muscle GP antibody corresponded to the brain isoform, although the lower brain/muscle protein content in patients may be due to the lack of normal muscle GP protein, as it occurs in muscle biopsies. Enzyme activity measurements revealed lower levels of the active form of GP activity and GP activity ratio, in patients' cell cultures compared to controls. Nevertheless, total GP activity was similar in both cell types, in agreement with previous reports [14], [16], [39]. GP isoenzymes exhibit differential regulatory features [41], [42]. Our data suggest that the lower levels of active GP in patients' cell cultures are not due to the liver isoenzyme: reduced liver isoenzyme, which is poorly activated by AMP, would be expected to cause similar decreases in the GP activity measured without AMP (active) or with AMP (total). This is not the case: total GP activity levels were comparable between patients' and controls' cultures. Consequently, the reduction should be due to lower AMP-sensitive muscle or brain GP isoenzyme activities. In summary, immunoblotting and enzyme activity data suggest that the reduction in GP protein and activity in patient's cultures corresponds to the muscle GP protein isoform.

With regard to the counterpart enzyme in glycogen metabolism, patients' cells showed lower levels of the active form of GS activity and of GS activity ratio, but equivalent total GS activity. Although GS has not been studied before in McArdle patients' cell cultures, previous research performed *in vivo* in the muscle of McArdle patients showed similar findings, i.e. preserved total GS activity levels compared to control subjects, but lower fractional velocity of GS in the basal condition, at the end of an insulin clamp and after exercise [43]. Overall these data suggest that GP does somehow enhance the activation of its opposite counterpart in glycogen metabolism. In this way, the lack of GP may, as a compensatory mechanism, contribute to inactivate GS and prevent glycogen overload in McArdle patients. In line with this hypothesis, in the patients' cultures, glycogen content compared to healthy controls' cultures was similar with a tendency to lower levels, as assessed by glycogen quantification in cell extracts, and lower number of glycogen granules with slightly larger diameter, as observed by electron microscopy. It should be considered that the smaller difference observed by quantification of glucose following the isolation and the hydrolysis of muscle cell glycogen may be due to variations in branch density [44] or glucose contamination [45] of glycogen or other factors that may affect this technique. Previous research showed similar glycogen content in McArdle and control muscle cultures [14]. *In vitro* data are in contrast to the *in vivo* findings of abnormal accumulation of glycogen. We hypothesize that *in vivo*, the much larger fall in GP activity in patient's muscle is likely undercompensated by GS inactivation. More research is necessary to clarify the GP mechanism of action and metabolic adaptations that McArdle cultures undergo in the *in vitro* condition.

No effective gene therapy is expected to be available in the foreseeable future to replace skeletal muscle GP [6]. Thus, a deeper knowledge of the regulation and the expression of the different GP isoforms is necessary, as it could contribute to the development of new therapeutic approaches for McArdle's disease. The re-expression of *PYGB* or *PYGL* in skeletal muscle could restore GP activity in this tissue. The “proof-of-principle” of such a strategy has been demonstrated by the ability of utrophin to compensate for the deficiency of dystrophin in the dystrophin-deficient mouse muscle [46], and by the ability of ε-sarcoglycan to compensate for the lack of α-sarcoglycan in autosomal recessive limb-girdle muscular dystrophy type-2D [47]. Because distinct GP isoforms are naturally produced by different tissues within the same patient, no rejection related problems are to be expected with this type of ‘re-expression’ therapy. Some problems could nevertheless arise from the fact that GP isoenzymes can differ in their regulatory properties and physiological role. Liver GP is activated in hepatocytes to maintain glucose homeostasis in the whole body, brain GP is activated in cases of cerebral anoxia and low glucose availability, whereas muscle GP functions as a glucose supplier only in working skeletal muscle fibers.

To conclude, we have reported a novel *PYGM* mutation, *p.R771PfsX33*, in two brothers with McArdle's disease, which causes reduction in *PYGM* mRNA, absence of protein and GP activity. The study of muscle cell cultures from these two patients and two controls has allowed us to find some clues as to the puzzling observation of GP-activity positive cultured cells derived from GP-activity negative muscle biopsy: (1) *PYGM* mRNA levels are strongly reduced in muscle cultured cells; (2) differences in the relative expression of brain and liver GP genes arise during differentiation in muscle cell culture; (3) most of the GP protein and activity in cultured muscle cells are brain and liver isoforms; (4) our data supports the notion that lack of muscle GP in McArdle's disease contributes to inactivate GS, thus preventing glycogen overload in muscle cultured cells.
